# Distribution and Potential Sources of OCPs and PAHs in Waters from the Danshui River Basin in Yichang, China

**DOI:** 10.3390/ijerph19010263

**Published:** 2021-12-27

**Authors:** Wei Chen, Bo Peng, Huanfang Huang, Ye Kuang, Zhe Qian, Wenting Zhu, Wei Liu, Yuan Zhang, Yuan Liao, Xiufang Zhao, Hong Zhou, Shihua Qi

**Affiliations:** 1 State Key Laboratory of Biogeology and Environmental Geology, China University of Geosciences, Wuhan 430078, China; wei.chen@cug.edu.cn (W.C.); pengbomh@163.com (B.P.); 20131001188@cug.edu.cn (Z.Q.); zhangyuan@cug.edu.cn (Y.Z.); 2 School of Environmental Studies, China University of Geosciences, Wuhan 430078, China; 3 Hubei Key Laboratory of Environmental Water Science in the Yangtze River Basin, China University of Geosciences, Wuhan 430078, China; 4 Institute of Geological Survey, China University of Geosciences, Wuhan 430074, China; kuangye@cug.edu.cn (Y.K.); zwt202110@163.com (W.Z.); zhouhong@cug.edu.cn (H.Z.); 5 State Key Laboratory of Organic Geochemistry, Guangzhou Institute of Geochemistry, Chinese Academy of Sciences, Guangzhou 510640, China; 6 South China Institute of Environmental Sciences, Ministry of Ecology and Environment, Guangzhou 510535, China; hhuanfang@outlook.com; 7 Geological Environmental Centre of Hubei Province, Wuhan 430034, China; liaoyuan18@aliyun.com; 8 7th Institute of Geology & Mineral Exploration of Shandong Province, Linyi 276000, China; zhxfwg@163.com

**Keywords:** groundwater, spring water, karstic river, distribution, mass flux

## Abstract

To investigate the concentrations, spatial distribution, potential sources and mass fluxes of organochlorine pesticides (OCPs) and polycyclic aromatic hydrocarbons (PAHs) in waters from the Danshui River Basin, a total of 20 water samples were collected and analyzed from a karstic river in Western Hubei of Central China. The average concentrations of total OCPs and PAHs in the river water were 4719 pg·L^−1^ and 26.2 ng·L^−1^, respectively. The characteristic ratios of different isomers and the composition analysis of individual OCPs and PAHs revealed that HCHs originated from a mixed input of technical HCHs and Lindane, DDTs were mainly from technical DDTs, and PAHs mainly originated from biomass and coal combustion. The mass flux analysis showed that PAHs had a higher emission and heavier burden than OCPs in the Danshui River Basin. OCPs and PAHs emitted from agricultural or other human activities could enter the groundwater and then be transported to the surface/river water in the karst area. The adsorption of OCPs and PAHs by particles and the sedimentation of particles could be the primary processes to intercept these pollutants in the water of the karstic river system.

## 1. Introduction

Organochlorine pesticides (OCPs) and polycyclic aromatic hydrocarbons (PAHs) are two typical groups of persistent organic pollutants (POPs). They have been widely studied due to their unique properties [1,2,3,4], such as persistence, long-range transport, toxicity and bioaccumulation. OCPs and PAHs present considerable mobility, and their occurrences have been widely reported on many regional occasions and even on the global scale over the past decades [2,5,6,7,8,9]. Due to their toxicity and long-term persistence in the environment, they threaten human health and ecological safety significantly [10].

In the karst area of Central China, anthropogenic activities were the major source of organic pollutants, such as OCPs and PAHs. The surface of the karst system, including the soil and the epikarst zone, is the most sensitive layer [11], can intercept and store these pollutants. However, due to its unique geological structure, the surface of the karst system is thin and even occasionally absent, and fractures and conduits are widely developed in this zone [12]. Thus, they can directly deliver the pollutants into groundwater, which may be the drinking water source for local residents, leading to the pollution of the underground systems and potential risks to human health.

Currently, most attention is still focused on the POPs in developed areas; few studies have been conducted in karst areas: Rodríguez et al. (2011) detected high concentrations of OCPs in karst groundwater in Yucatan, Mexico [13]. Schwarz et al. (2011) found that PAHs could be effectively retained in the soil even in a highly vulnerable karst catchment unless extremely high discharge events occurred [14]. Perrette et al. (2013) demonstrated that PAHs could directly transfer from the atmosphere into the seepage waters through the depositions in the shallow Elaphes cave in France [15]. Levy et al. (2017) figured out that the transport of the most soluble PAHs in the karst system was predominant, and environmental karstic conditions could significantly affect the potential degradation and stability of OCPs in the Alps karst system in Zugspitze, Germany [16]. Lan et al. (2016) identified that the wastewater discharge and surface water leakage were the major sources of PAHs in karst groundwater and sediments from in the Laolongdong underground river system, Chongqing, Southwest China [17]. Sun et al. (2020) showed that PAHs in the soil could be dissolved in soil seepage water and transported vertically downward into groundwater in the same system mentioned above [18]. Sun et al. (2019) found that epikarst spring water was contaminated by PAHs due to the loss of protection effects of soils in Nanchuan District, Chongqing, Southwest China [19]. However, there is still a lack of relevant field research on the migration of POPs at a whole karst watershed scale, from epikarst water or groundwater to surface water in the river.

The Danshui River is a tributary of the Qing River (called ‘Qingjiang’ in Chinese) in Yichang, Western Hubei. It belongs to a typical karst trough zone of China, which is characterized by a steep terrane of middle-low mountains and deep ravines [20]. In this area, the interconversion of surface water and groundwater takes place frequently [21]. Additionally, mountain agriculture has been highly developed in these karst depression areas with high elevation for more than three decades [22]. Thus, the Danshui River Basin could be a good area for studying the migration of organic pollutants, e.g., PAHs and OCPs, on a whole karst river basin scale. In this paper, we aim to (1) detect the concentrations and distribution of OCPs and PAHs in waters from the whole Danshui River, (2) discuss potential sources of OCPs and PAHs in waters and (3) estimate the mass flow of OCPs and PAHs in the river.

## 2. Materials and Methods

### 2.1. Study Area and Sampling

**Figure 1:**
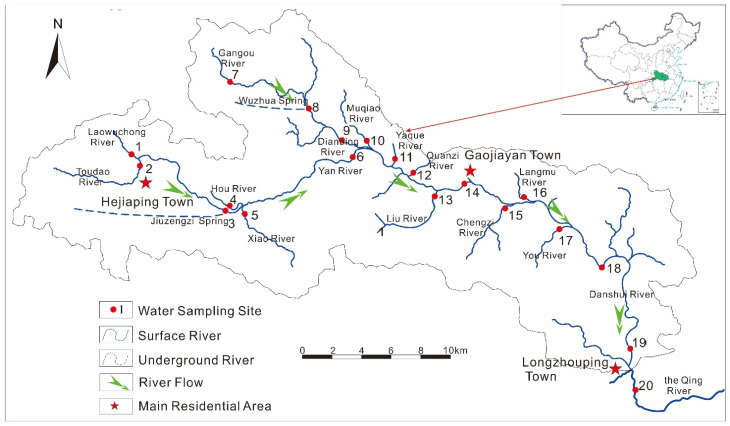
The location and sampling sites in the Danshui River.

The Danshui River Basin is located in Changyang County, Yichang City, West of Hubei Province, Central China (Figure 1). The upper stream is in the west section of the river (Sites 1–10), and its average altitude is around 1300 m, much higher than that of the downstream. Numerous karst depressions in high mountains have been reclaimed to grow mountain vegetables for more than three decades [22]. The pollutants from the agricultural activities could be delivered with the water flow from surface soil to groundwater and to surface river water.

To reveal the distribution and migration of OCPs and PAHs in the Danshui River Basin, river water (including two samples from underground rivers, Site 3 and Site 8 from Jiuzengzi Spring and Wuzhua Spring, respectively) was collected along the river. A total of twenty sites were selected (Figure 1) for water sampling. The sampling campaign was conducted in October 2019. At the same time, a hand-held current meter (flow tracker) was used to measure the water velocity (YSI, Yellow Springs, OH, USA). Water samples were collected in situ by amber glass bottles without bubbles after being rinsed with water three times. Afterward, they were transported to the laboratory and stored in a refrigerator at 4 °C before pretreatment within 7 days.

### 2.2. Sample Pretreatment and Instrumental Analysis

The water samples were pretreated following the method of previous studies in the same research group with minor modification [23,24]. Each water sample (2 L) was added to a separation funnel and spiked with recovery surrogates (2,4,5,6-tetrachloro-*m*-xylene (TC*m*X) and polychlorinated biphenyls-209 (PCB209) for OCPs; naphthalene-d_8_ (Nap-d_8_), acenaphthene-d_10_ (Ace-d_10_), phenanthrene-d_10_ (Phe-d_10_), chrysene-d_12_ (Chr-d_12_) and perylene-d_12_ (Per-d_12_) for PAHs). Water samples were then extracted by liquid-liquid extraction with dichloromethane (DCM) three times (50 mL of DCM was added each time). After extraction, extracts were collected in a flat-bottomed flask and were dehydrated by anhydrous sodium sulfate. Each extract was concentrated, solvent exchanged to *n*-hexane and then reduced to ca. 3–5 mL on a rotary evaporator (G3, Heidolph, Schwabach, Germany). The concentrates were passed through chromatography columns containing deactivated silica gel and alumina (*v*:*v* = 2:1) for purification and then eluted with a mixture of DCM and *n*-hexane (*v*:*v* = 2:3). Each eluate was concentrated by the rotary evaporator and was reduced to 0.2 mL under a gentle flow of high-purity nitrogen (>99.999%). Then, 20 ng of pentachloronitrobenzene (PCNB) and 1000 ng of hexamethylbenzene (HMB) were spiked as internal standards into the sample for OCP and PAH analysis, respectively. The samples were stored at −20 °C until instrumental analysis.

Twenty-four OCPs, including four hexachlorocyclohexane (HCH) isomers (*α*-, *β*-, *γ*- and *δ*-HCH), six dichloro-diphenyl-trichloroethane (DDT) isomers and their derivatives (*o,p*′-DDE *p,p*′-DDE, *o,p*′-DDD, *p,p*′-DDD, *o,p*′-DDT and *p,p*′-DDT), hexachlorobenzene (HCB), aldrin, dieldrin, endrin, endrin aldehyde, endrin ketone, methoxychlor, heptachlor, heptachlor epoxide, *cis*-chlordane (CC), *trans*-chlordane (TC), *α*-endosulfan (*α*-Endo), *β*-endosulfan (*β*-Endo) and endosulfan sulfate (ES), were quantitatively analyzed with a gas chromatograph (7890A, Agilent, Santa Clara, CA, USA) equipped with a ^63^Ni- electron capture detector (GC-ECD) [23]. Sixteen PAHs, which were listed by the U.S.EPA as priority PAHs, including naphthalene (Nap), acenaphthylene (Acy), acenaphthene (Ace), fluorene (Flu), phenanthrene (Phe), anthracene (Ant), fluoranthene (Fla), pyrene (Pyr), benzo(a)anthracene (BaA), chrysene (Chr), benzo(b)fluoranthene (BbF), benzo(k)fluoranthene (BkF), benzo(a)pyrene (BaP), indeno(1, 2, 3-cd)pyrene (IcdP), dibenzo(a, h)anthracene (DahA) and benzo(g, h, i)perylene (BghiP), were analyzed by gas chromatography–mass spectrometry (GC-MS, Agilent 7890 N GC-5975MSD) [24]. The detailed settings of parameters for GC-ECD and GC-MS analysis have been well described in previous studies [23,24,25]. The chromatograms of the standards and typical samples for OCP and PAH analyses are shown in Figure S1, respectively.

### 2.3. Quality Assurance and Quality Control

Internal standard methods were applied to establish the response factor calibration curves to quantify the target OCPs and PAHs. The ranges of the linearity and linearity correlation coefficients (*R*^2^) of the calibration curves are listed in Table S1. The procedure blanks, parallel samples and recovery surrogates were used for quality control (QC) during sample processing. Specifically, during sample pretreatment, the procedure blanks and parallel samples were treated with the same procedure as normal samples, and recovery surrogates were added in every sample to correct the possible impact during the sample pretreatment on the results. During instrumental analysis, after daily cleaning with *n*-hexane (solvent blank), a QC standard solution was tested to make sure the variation in results was less than 10% compared to the standard curve of the targets on that day. There were no target compounds detected in the procedure blanks and solvent blanks, and the relative standard deviations (RSDs) were within 20% for the parallel samples. Method detection limits (MDLs) of OCPs and PAHs in the water were calculated based on three times of the signal/noise ratio, which ranged from 10 to 20 pg·L^−1^ and from 0.02 to 1.80 ng·L^−1^, respectively (Table S1). The recoveries (mean ± standard deviation) for OCPs were 66 ± 14% and 92 ± 13% for TC*m*X and PCB209, respectively; for PAHs, recoveries were 28 ± 11%, 56 ± 11%, 91 ± 17%, 89 ± 29% and 107 ± 28% for Nap-d_8_, Ace-d_10_, Phe-d_10_, Chr-d_12_ and Per-d_12_, respectively.

## 3. Results and Discussion

### 3.1. Concentrations and Spatial Distribution of OCPs and PAHs

#### 3.1.1. Concentrations and Spatial Distribution of OCPs

OCP compounds were ubiquitous in the water of the Danshui River Basin (Table 1). The concentrations of ∑_24_OCPs (the sum of 24 OCPs) in the water ranged from 1225 to 31,225 pg·L^−1^, with an average (avg.) of 4719 ± 6794 pg·L^−1^. HCHs and DDTs are two of the most important OCP groups; the concentrations of ∑_4_HCHs (sum of four HCH isomers) and ∑_6_DDTs (sum of six DDTs and their derivatives) were 104–20,931 pg·L^−1^ (avg. 1394 ± 4490 pg·L^−1^) and 116–3268 pg·L^−1^ (avg. 841 ± 680 pg·L^−1^), respectively. The higher average concentration of HCHs may be caused by the higher water solubility of HCHs than that of DDTs, so HCHs are more prone to migrate from soil/sediments to water than DDTs during the water flow with surface runoff in the karst area [25], which leads to larger variations in HCHs (can be characterized by the coefficient of variation (CV), 322% for HCHs and 81% for DDTs). Accounting for 17.2, 10.6 and 10.1% of ∑_24_OCPs, respectively, *β*-HCH, aldrin and *β*-Endo were the most abundant compounds (concentration range: 29–12,936 pg·L^−1^, 17–3427 pg·L^−1^ and <MDL–8493 pg·L^−1^, respectively), while *β*-HCH, HCB and aldrin were the most prevalent compounds (detection rate: 100%, Table 1).

Compared with other areas, the concentrations of ∑_4_HCHs (avg. 13.9 ng·L^−1^) and ∑_6_DDTs (avg. 0.84 ng·L^−1^) in the Danshui River Basin were lower than those of the Dong Nai River System in Vietnam (140 and 120 ng·L^−1^) [5]; the Tonghui River of Beijing, China (254 and 155 ng·L^−1^) [26]; and the Yongding River basin, China (4.03 ng·L^−1^ and 3.89 ng·L^−1^) [27]. These concentrations are similar to those found in the Pyrenees (2.9 and 0.016 ng·L^−1^) [28] and higher than those in the Alps (0.99 and 0.014 ng·L^−1^) [28] and the Himalayan region (<0.02 ng·L^−1^) [29]. These comparisons indicated the low–medium level of water OCPs in the study area.

**Figure 2:**
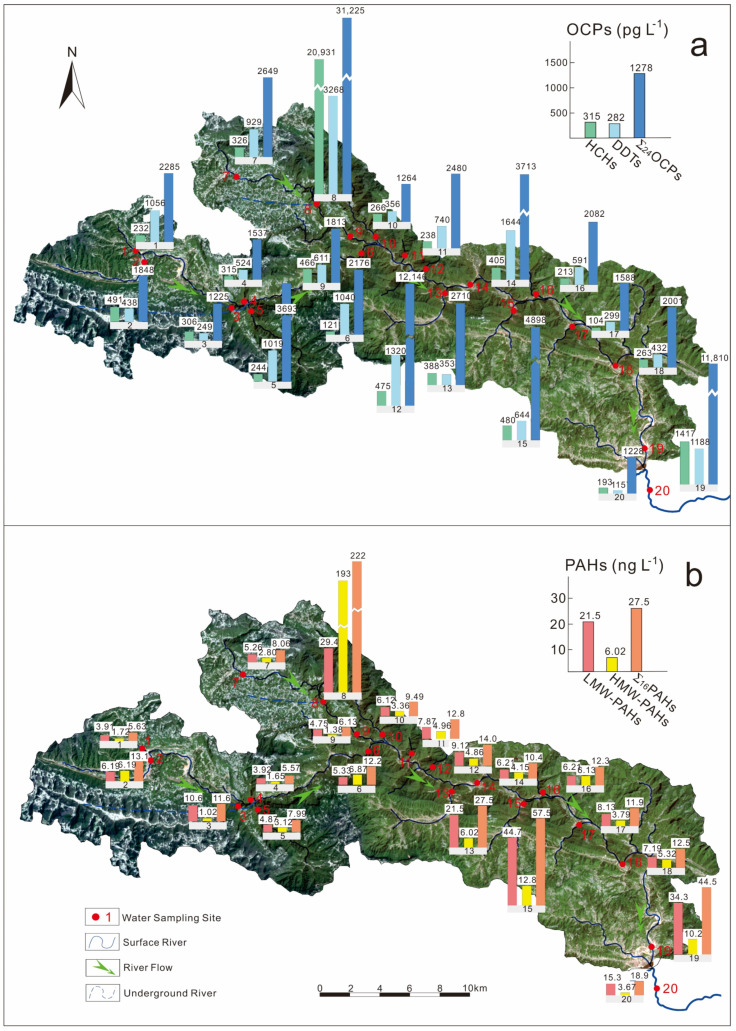
Spatial distribution of OCPs (**a**) and PAHs (**b**) in the Danshui River.

The concentrations of OCPs were highly varied (Figure 2a) along the Danshui River basin, indicated by the high coefficients of spatial variations for ∑_24_OCPs (CV, 144%) and individual OCP compounds (CV: 69.9–386%). High OCP concentrations were observed at Sites 8, 12, 15 and 19, with concentrations of ∑_24_OCPs, ∑_4_HCHs and ∑_6_DDTs ranging from 4898 to 31,225 pg·L^−1^, 475 to 20,931 pg·L^−1^ and 644 to 3268 pg·L^−1^, respectively. The Danshui River Basin is divided into four sections for further discussion: (1) Upstream 1, the Hou River with sampling Sites 1–6, with waters from these sites mostly originating from underground, especially Site 3 (the Jiuzengi Spring); (2) Upstream 2, the Dianbing River with Sites 7–10, which has a similar situation to the Hou River, and Site 8 is the Wuzhua Spring; (3) Middle Stream, from Sanyouping to Gaojiayan Town with Sites 11–17; and (4) Downstream, from Gaojiayan Town to the outlet to the Qing River with Sites 18–20 (Figure 1). Overall, the average concentrations of ∑_24_OCPs increased from the upstream (2126 ± 862 pg·L^−1^ for Upstream 1) to the middle stream (4231 ± 3659 pg·L^−1^) and downstream (5013 ± 5899 pg·L^−1^), except for those of Upstream 2 (9237 ± 14669 pg·L^−1^) due to the extremely high concentration at Site 8. This indicated the transport and accumulation of OCPs along the river. The concentrations of ∑_24_OCPs were the highest at Site 8 (Figure 2a), especially for HCHs, which may be caused by the supply from the discharge area where there are mountain vegetable farm lands [22] or/and the surrounding point source pollution [30]. In the past, mountain vegetable agriculture lands applied large amounts of pesticides. Most compounds were relatively high at Sites 15 and 19, near the main residential areas (Figure 1). These pollutants may be emitted by intensive human activities (such as the farming and domiciliary applications of OCPs in the past) [31] from local residents.

#### 3.1.2. Concentrations and Spatial Distribution of PAHs

The concentrations of ∑_16_PAHs (sum of 16 PAHs) ranged from 5.49 to 222 ng·L^−1^ (avg. 26.2 ng·L^−^^1^, Table 2) in the water of the Danshui River Basin, with most sites having less than 20 ng·L^−1^, except Sites 8 (222 ng·L^−1^), 13 (27.5 ng·L^−1^), 15 (57.5 ng·L^−1^) and 19 (44.5 ng·L^−1^). The concentrations of the low-molecular-weighted PAHs (two to three rings, LMW-PAHs, range: 3.91–44.7 ng·L^−1^, avg. 12.0 ng·L^−1^) were lower than those of the high-molecular-weighted PAHs (four to six rings, HMW-PAHs, range: 1.02–193 ng·L^−1^, avg. 14.2 ng·L^−1^). However, the detection rates of the LMW-PAHs (100%) were higher than those of the HMW-PAHs (95%) due to the LMW-PAHs having lower octanol–water partition coefficients and weaker hydrophobicity than those of HMW-PAHs [27]. The most abundant PAH compounds were Pyr (8.50 ng·L^−1^), Nap (5.74 ng·L^−1^), Phe (4.33 ng·L^−1^), Fla (2.24 ng·L^−1^), BbF (1.46 ng·L^−1^), BaP (1.37 ng·L^−1^) and Flu (1.24 ng·L^−1^). Among these compounds, Nap, BbF and BaP were detected in all samples from the river water.

Compared with other regions, the ∑_16_PAH concentrations in the Danshui River (avg. 26.2 ng·L^−1^) were much lower than those in the Minjiang River Estuary, China (avg. 72,400 ng·L^−1^) [32]; slightly lower than those in the Yongding River, China (avg. 124 ng·L^−1^) [27]; and higher than those in the Himalayan region (1.90 ng·L^−1^) [29].

Similar to the distribution of OCPs, the variation in the PAH concentrations along the river is relatively high (CV: 138%). The average concentrations of LMW-PAHs, HMW-PAHs and ∑_16_PAHs increased from the upstream (5.80 ± 2.50 ng·L^−1^, 3.55 ± 2.68 ng·L^−1^ and 9.35 ± 3.39 ng·L^−1^ for Upstream 1, respectively) to the middle stream (14.8 ± 14.2 ng·L^−1^, 6.11 ± 3.07 ng·L^−1^ and 20.9 ± 17.1 ng·L^−1^, respectively) and downstream (18.9 ± 13.9 ng·L^−1^, 6.39 ± 3.38 ng·L^−1^ and 25.3 ± 16.9 ng·L^−1^, respectively), except for those of Upstream 2 (11.4 ± 12.0 ng·L^−1^, 50.1 ± 95.2 ng·L^−1^ and 61.5 ± 107 ng·L^−1^, respectively) due to the same extremely high concentration at Site 8 (Figure 2b). This showed that water flow is the important carrier for PAH transport. At Site 8, of the concentrations of most individual PAH compounds were higher than those at other sites. This phenomenon was similar to that of OCPs, indicating the point source pollutions at this site. At the same time, a high correlation coefficient was observed between concentrations of ∑_16_PAHs and ∑_24_OCPs (*R*^2^ = 0.595, *p* < 0.01), especially for HMW-PAHs, suggesting a high consistency of the sources of PAHs and OCPs in the basin, which could be the intensive anthropogenic activities.

### 3.2. Source Diagnosis of OCPs and PAHs

#### 3.2.1. Potential Source Analysis of OCPs

**Figure 3:**
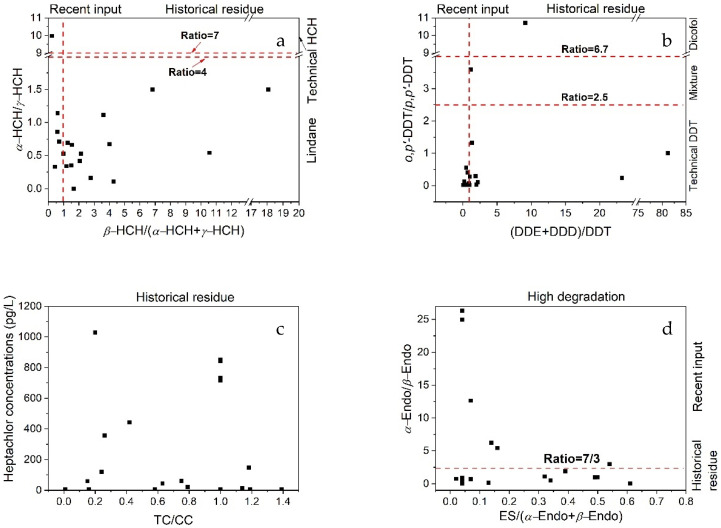
Ratios of HCHs (**a**), DDTs (**b**), chlordane (**c**) and endosulfan (**d**) in the Danshui River.

HCHs in the environment mainly come from technical HCHs and Lindane. Technical HCHs are composed of *α*-HCH (55–80%), *β*-HCH (5–14%), *γ*-HCH (8–15%) and *δ*-HCH (2–16%), while Lindane mainly contains *γ*-HCH (99%) [23]. *α*-HCH/*γ*-HCH ratios of <4 and 4–7 could indicate the fresh application of Lindane and technical HCHs, respectively. A ratio of >7 indicates the historical use of technical HCHs since *γ*-HCH can transform to *α*-HCH through long-term weathering after application [33]. In this study, all ratios of *α*-HCH/*γ*-HCH (except Site 14) were less than 4 (avg. 0.47, Figure 3a), which suggested the prevalent fresh input of Lindane in the study area. In addition, *α*-HCH and *γ*-HCH could be transformed into *β*-HCH through long-term weathering. Thus, the ratio of *β*-HCH/(*α*-HCH + *γ*-HCH) can reflect the recent input or historical residues of technical HCHs. A high average concentration (811 pg·L^−1^) of *β*-HCH with a 95% detection rate (Table 1) was observed, showing a higher stability and a weaker degradation and metabolic capacity of *β*-HCH than those of the other HCH isomers [34,35]. The ratios of *β*-HCH/(*α*-HCH + *γ*-HCH) ranged from 0.23 to 12.1 (avg. 3.25, Figure 3a) in the river water. Ratios for most samples were >1, indicating that HCHs had been highly degraded, which suggested that they were residues of the technical HCHs. These two ratios indicated that the recent application of Lindane and the historical residues of technical HCHs are the main sources of HCHs in the Danshui River Basin.

Technical DDTs contain *p,p*′-DDT (80–85%) and *o,p*′-DDT (15–20%), which are different to those in commercial dicofol (1.7% *p,p*′-DDT and 11.4% *o,p*′-DDT) [36]. Thus, the *o,p*′-DDT/*p,p*′-DDT ratio can be used to determine the source of DDTs. *o,p*′-DDT/*p,p*′-DDT ratios of <2.5 and >6.7 suggest the sources are technical DDTs and dicofol, respectively [37,38]. In this study, *o,p*′-DDT/*p,p*′-DDT ratios ranged from 0 to 3.58 (Figure 3b), with low ratios (<2) found in 90% of samples, indicating the main source of technical DDTs. In only two samples, ratios fell within the range of 3.59–10.7, suggesting the existence of a dicofol source at these sites. DDT can be degraded into DDE under aerobic conditions and into DDD under anaerobic conditions by microorganisms [39]. Therefore, the ratios of DDE/DDD and (DDE + DDD)/DDT could be used to determine the degradation conditions (aerobic or anaerobic) and the sources from new or historical DDT input [23]. That is, if the ratio of DDE/DDE is larger than 1, it represents an aerobic environment; otherwise, it represents an anaerobic environment [40]. If the ratio of (DDE + DDD)/DDT is larger than 1, it represents historical residue; otherwise, it is a recent input [41]. In this study, the ratios of DDE/DDD were mostly less than 1, showing an anaerobic degradation environment, which indicated that the groundwater could be an important transport intermediary of the DDTs into the river water of the Danshui River. The ratios of (DDE + DDD)/DDT were close to or larger than 1 (avg. 6.40, Figure 3b) at most sites, which indicated that the historical residue of technical DDTs is the main source of DDTs in the Danshui River.

Some other characteristic ratios, such as *trans*-chlordane/*cis*-chlordane (TC/CC) and *α*-endosulfan/*β*-endosulfan (*α*-Endo/*β*-Endo), were also applied to diagnose the sources of chlordane and endosulfan. The ratio of TC/CC in technical chlordane is close to 1.56 [42] since the technical chlordane in the international market contains 13% TC, 11% CC, and 5% heptachlor. The TC degrades faster than CC in the environment. Thus, it is expected that the ratio of TC/CC is less than 1.56 in the natural environment, and it represents the historical residue of technical chlordane; otherwise, it is a recent input or due to other sources. In the Danshui River, the ratios were all less than 1.4 (Figure 3c), indicating the historical residue of technical chlordane, which was similar to the results from the karst spring systems [43] and in the soils [38] near the Three Gorges Dam.

Endosulfan was widely used in China until March 2019. Usually, commercial endosulfan contains *α*-Endo and *β*-Endo in the ratio of 7:3 [31]. Both isomers can be degraded into ES in the environment, while α-Endo is less persistent and more volatile compared with *β*-Endo [44]. Thus, the ratio of *α*-Endo/*β*-Endo can represent the historical residue or recent input of commercial endosulfan when the ratio is >7/3 or <7/3, respectively. The ratios of ES/(*α*-Endos + *β*-Endo) can represent the degradation degree. A ratio larger than 1 means high degradation. In the Danshui River water, the detection rate of ES was only 35%, which was much lower than that of *α*-Endo and *β*-Endo, with all the ratios of ES/(*α*-Endo + *β*-Endo) <1, showing a low degradation and relatively new sources of endosulfan (Figure 3d). At the same time, 30% of the ratios of *α*-Endo/*β*-Endo were larger than 1, with these sites distributed from the upstream to the downstream, indicating a relatively fresh input of endosulfan in the Danshui River Basin.

#### 3.2.2. Potential Source Analysis of PAHs

PAHs in the environment mainly come from petrogenic sources and incomplete combustions of petroleum and other fossil fuels, as well as coal and biomass. Incomplete combustions can come from both anthropogenic and natural processes, and the former is gradually becoming the major contributor [45]. The ratios between the characteristic compounds of PAHs can be applied to identify potential source patterns [46]. Two ratios of Fla/(Fla + Pyr) and BaA/(BaA + Chr) were widely applied. Fla/(Fla  +  Pyr) values  <0.4, between 0.4 and 0.5 and >0.5, indicate a petroleum source, fossil fuel combustion, and biomass and coal combustion, respectively [46]. Similarly, if the ratio of BaA/(BaA + Chr) is <0.2, between 0.2 and 0.35 and >0.35, it represents the contribution of petroleum/petrogenic sources, mixed sources and combustion, respectively [46].

**Figure 4:**
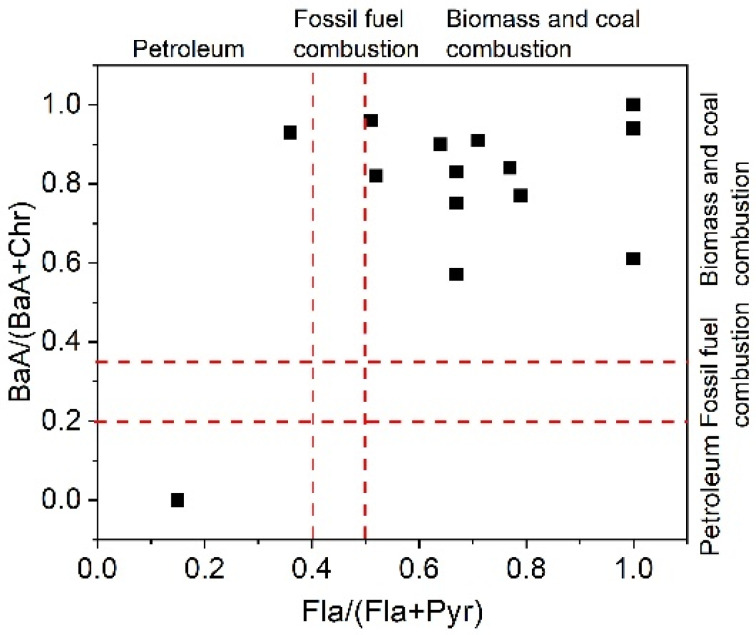
Ratios of PAH compounds in the Danshui River.

In this study, the ratios of Fla/(Fla  +  Pyr) at most sites were higher than 0.5 (Figure 4), excluding Site 8 (<0.4), which indicated the preponderance of biomass and coal burning in this area. This was also confirmed by the BaA/(BaA + Chr) ratios (more than 0.35 at most sites, Figure 4). This is consistent with the characteristics of the local energy consumption in the remote mountain area. Only Site 8 differs, showing a petroleum source. Combining the discussion with Section 3.1, point source pollution, such as waste disposal, could be possible.

### 3.3. Mass Flows of OCPs and PAHs

**Figure 5:**
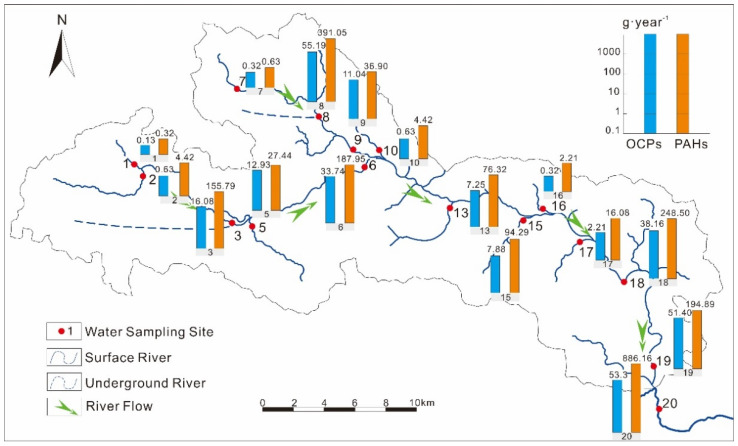
Mass fluxes (g·year^−1^) of OCPs and PAHs along the Danshui River.

River flow rates at different sections were determined by a hand-held flow tracker. The mass fluxes of OCPs and PAHs were calculated by the measured concentrations and flow rates (Table S2). The mass fluxes of OCPs and PAHs (Figure 5) in the upstream were 131 and 809 g·year^−1^, respectively; 17.7 and 189 g·year^−1^ in the middle section, respectively; and 53.3 and 886 g·year^−1^ in the downstream, respectively. There were much higher mass flows of PAHs than of OCPs, showing the higher emission and heavier burden of PAHs in the Danshui River Basin. Relatively high flow fluxes of OCPs and PAHs in the upstream indicated that the karst groundwater is an important transport intermediary of these pollutants: OCPs and PAHs emitted from agricultural or other human activities in the karst river basin can easily enter the groundwater and are then discharged to the surface water through spring water or to the hyporheic flow in the river, such as in the Danshui River, since these flows are the main sources of the surface water in the upstream of the river in the karst area. This also confirmed the discussion in Section 3.2.1. Potential source analysis of OCPs. Additionally, the adsorption of OCPs and PAHs by particles and the sedimentation of particles could be the main processes to intercept the POPs in the water from the karstic surface system. This is reflected in the proportions of LMW-PAHs and HMW-PAHs from the upstream to the downstream (Figure S2): the proportion of LMW-PAHs was less than 50% in the upstream but was more than 80% in the middle stream and downstream.

## 4. Conclusions

OCPs and PAHs were ubiquitous in the water of the karstic river—the Danshui River. Their concentrations were much lower than those in developed industrial and agricultural areas but higher than those in remote and less developed areas. HCHs in the Danshui River water originated from the mixed input of technical HCHs and Lindane. DDTs mainly came from the input of technical DDTs. Chlordane mainly came from the historical residue of technical chlordane, while a relatively fresh input of endosulfan might exist in the Danshui River Basin. PAHs in this area mainly came from biomass and coal combustion due to the less developed socio-economic status in the Danshui River Basin. Point source pollution might happen in the Wuzhua Spring (Site 8). OCPs and PAHs emitted from agricultural or other human activities could be transported from the groundwater to the surface water in the karstic Danshui River Basin. The adsorption of OCPs and PAHs by particles and the sedimentation of particles might be the main processes to intercept the POPs in the water of the karstic river system.

## Figures and Tables

**Table 1:** Concentrations (pg·L^−1^) and detection rates (%) of OCPs in the Danshui River.

Compounds	Range	Mean ± SD	Median	Detection Rates
*α*-HCH	<MDL–253	50.5 ± 59.5	28.6	65
*β*-HCH	29.4–12,936	811 ± 2783	173	100
*γ*-HCH	<MDL–7743	455 ± 1673	50.8	90
*δ*-HCH	<MDL–546	76.6 ± 125	33.5	85
*o,p*′-DDE	<MDL–736	177 ± 227	76.4	70
*p,p*′-DDE	<MDL–110	20.3 ± 26.2	<MDL	45
*o,p*′-DDD	<MDL–1369	215 ± 385	25.9	45
*p,p*′-DDD	<MDL–589	68.0 ± 127	23.9	55
*o,p*′-DDT	<MDL–1180	93.8 ± 255	<MDL	40
*p,p*′-DDT	<MDL–561	266 ± 186	258	90
HCB	18.3–2716	431 ± 753	85.1	100
TC	<MDL–1315	102 ± 285	14.1	60
CC	<MDL–2075	173 ± 447	33.9	80
*α*-Endo	<MDL–234	53.0 ± 58.1	31.0	65
*β*-Endo	<MDL–8493	476 ± 1841	32.1	60
heptachlor	<MDL–1027	274 ± 347	59.5	70
heptachlor-epoxide	<MDL–956	151 ± 264	37.3	75
aldrin	16.5–3427	500 ± 729	350	100
dieldrin	<MDL–98.5	24.4 ± 27.5	<MDL	45
endrin	<MDL–657	54.2 ± 141	<MDL	45
endrin aldehyde	<MDL–199	44.4 ± 54.4	<MDL	45
endrin ketone	<MDL–110	23.4 ± 32.1	<MDL	20
ES	<MDL–338	45.2 ± 87.1	<MDL	35
methoxychlor	<MDL–1422	133 ± 312	<MDL	30
∑_4_HCHs	104–20,931	1394 ± 4490	310	100
∑_6_DDTs	116–3268	841 ± 680	628	95
∑_24_OCPs	1225–31,225	4719 ± 6794	2230	100

**Table 2:** Concentrations (ng·L^−1^) and detection rates (%) of PAHs in the Danshui River.

Compounds	Aromatic Ring	Range	Mean ± SD	Median	Detection Rate
Nap	2	2.76–12.1	5.74 ± 2.59	5.06	100
Acy	3	<MDL–1.22	0.33 ± 0.31	0.24	90
Ace	3	<MDL–1.69	0.37 ± 0.38	0.27	85
Flu	3	<MDL–6.73	1.24 ± 1.86	0.45	85
Phe	3	0.20–23.3	4.33 ± 6.50	1.01	100
Ant	3	<MDL–0.08	<MDL	<MDL	25
Fla	4	<MDL–28.9	2.24 ± 6.29	0.15	80
Pyr	4	<MDL–161	8.50 ± 35.0	0.06	55
BaA	4	<MDL–0.86	0.41 ± 0.17	0.36	95
Chr	4	<MDL–0.41	0.08 ± 0.09	0.05	80
BbF	5	0.17–3.04	1.46 ± 0.84	1.41	100
BkF	5	<MDL–1.03	<MDL	<MDL	10
BaP	5	<MDL–4.02	1.37 ± 1.01	1.13	90
Icdp	5	<MDL–0.08	<MDL	<MDL	10
DahA	6	<MDL	<MDL	<MDL	5
BghiP	6	<MDL–0.06	<MDL	<MDL	10
LMW-PAHs	2–3	3.91–44.7	12.0 ± 11.2	6.70	100
HMW-PAHs	4–6	1.02–193	14.2 ± 41.1	4.51	95
∑_16_PAHs	/	5.49–222	26.2 ± 46.8	12.3	100

## Data Availability

The data that support the findings of this study are available from the corresponding author, W. Liu (wliu@cug.edu.cn), upon reasonable request.

## Supplementary Materials

The following are available online at https://www.mdpi.com/article/10.3390/ijerph19010263/s1, Table S1: Method detection limits (MDLs), linearity range and linearity correlation coefficients (*R*^2^) of OCPs and PAHs; Table S2: Flow velocity (L·s^−1^) in the Danshui River Basin, Figure S1: Chromatograms of standard (a) and typical sample (b) for OCP analysis by GC-ECD and chromatograms of standard (c) and typical sample (d) for PAH analysis by GC-MS; Figure S2: Proportion of LMS-PAHs and HMW-PAHs in the different sections (US1: Upstream 1, US2: Upstream 2, MS: Middle Stream, DS: Downstream); data from Site 8 were not included due to the extremely high concentration.
