# Supplementary material for: Distribution and Potential Sources of OCPs and PAHs in Waters from the Danshui River Basin in Yichang, China
Source: Int J Environ Res Public Health. 2021 Dec 27;19(1):263. doi: 10.3390/ijerph19010263 (PMC8782434; doi:10.3390/ijerph19010263)
Supplement: Supplementary file 1 [file ijerph-19-00263-s001.zip › ijerph-1497278-supplementary.pdf]

Table S1. Method detection limits (MDLs), linearity range and linearity correlation coefficients (R^2) of OCPs and PAHs.

Compounds	MDLs (pg·L^{-1})	Linearity range (μg/L)	R^2	Compounds	MDLs (ng·L^{-1})	Linearity range (mg/L)	R^2
α-HCH	15	10–200	0.9999	Nap	1.8	0.05–10	0.9985
β-HCH	15	10–200	0.9998	Acy	0.04	0.05–10	0.9989
γ-HCH	10	10–200	0.9999	Ace	0.05	0.05–10	0.9985
δ-HCH	10	10–200	0.9998	Flu	0.04	0.05–10	0.9993
o,p′-DDE	10	10–200	0.9999	Phe	0.13	0.05–10	0.9993
p,p′-DDE	10	10–200	0.9999	Ant	0.02	0.05–10	0.9995
o,p′-DDD	10	10–200	0.9999	Fla	0.02	0.05–10	0.9995
p,p′-DDD	10	10–200	0.9999	Pyr	0.05	0.05–10	0.9996
o,p′-DDT	20	10–200	0.9999	BaA	0.1	0.05–10	0.9993
p,p′-DDT	20	10–200	0.9999	Chr	0.02	0.05–10	0.9991
HCB	12	10–200	0.9996	BbF	0.1	0.05–10	0.9992
methoxychlor	20	10–200	0.9974	BkF	0.05	0.05–10	0.9990
TC	10	10–200	0.9999	BaP	0.2	0.05–10	0.9994
CC	10	10–200	0.9999	Icdp	0.02	0.05–10	0.9973
heptachlor	13	10–200	0.9999	DahA	0.02	0.05–10	0.9988
heptachlor-epoxide	20	10–200	0.9999	BghiP	0.02	0.05–10	0.9992
α-Endo	10	10–200	0.9999	/	/	/	/
β-Endo	10	10–200	0.9998	/	/	/	/
ES	10	10–200	0.9998	/	/	/	/
aldrin	15	10–200	0.9999	/	/	/	/
dieldrin	15	10–200	0.9993	/	/	/	/
endrin	15	10–200	0.9992	/	/	/	/
endrin aldehyde	20	10–200	0.9998	/	/	/	/
endrin ketone	20	10–200	0.9999	/	/	/	/

Table S2. Flow velocity (L·s^{-1}) in the Danshui River Basin.

Sample	Flow velocity
1	2
2	11
3	430
5	110
6	489
7	2
8	56
9	193.4
10	14.8
13	88
15	52
16	5.9
17	43
18	638
19	139
20	1488

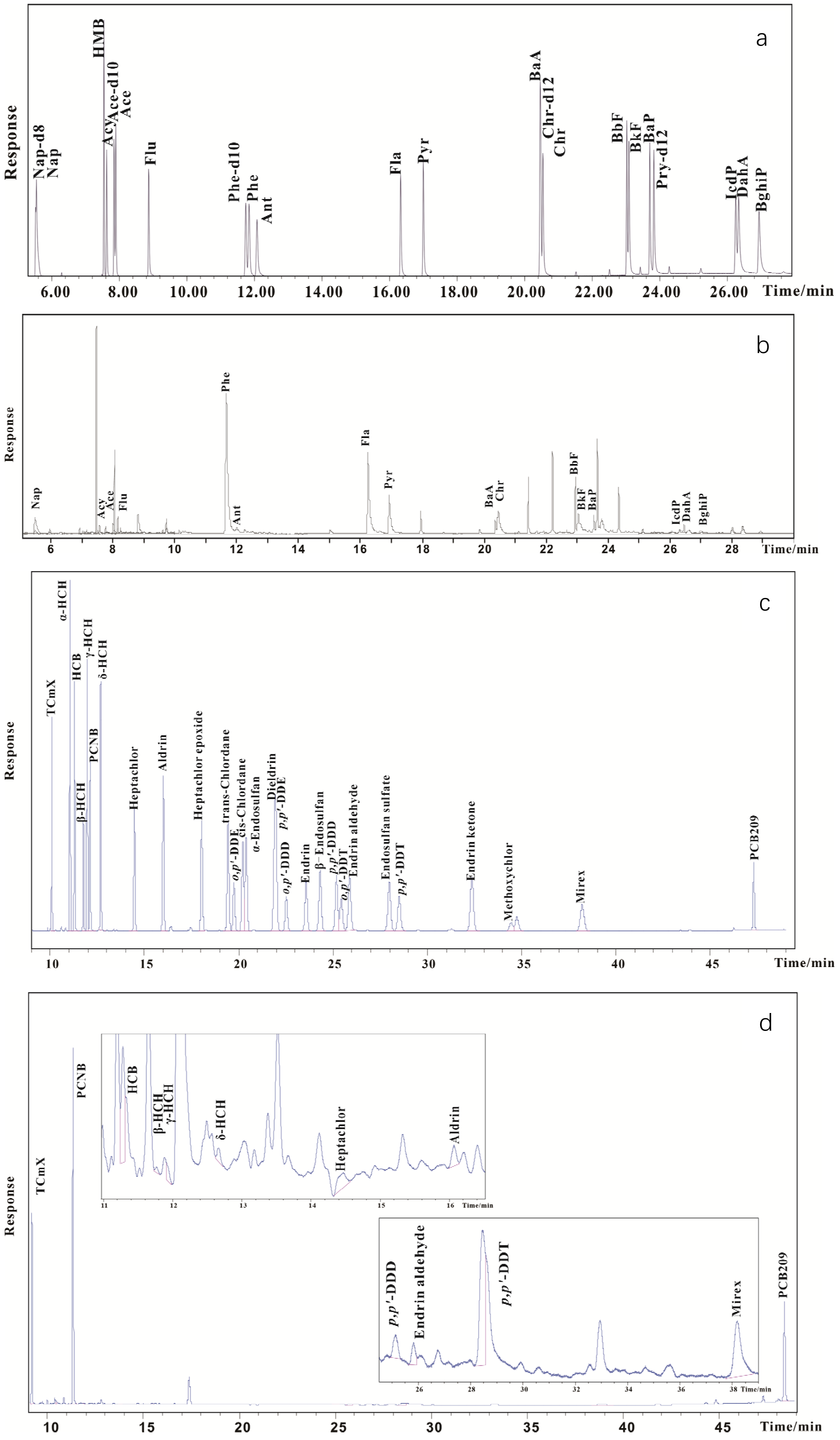

Figure S1. Chromatograms of standard (a) and typical sample (b) for OCPs analysis by GC-ECD and chromatograms of standard (c) and typical sample (d) for PAHs analysis by GC-MS

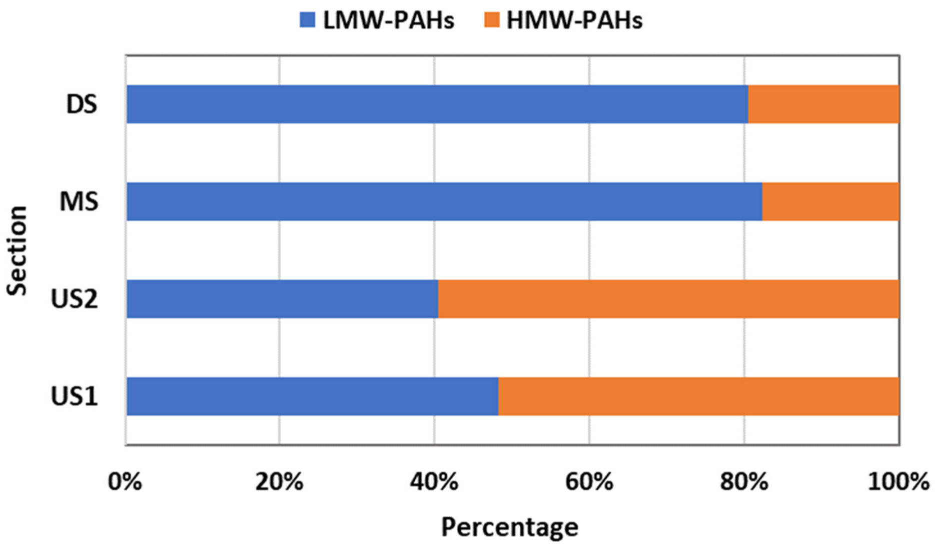

Figure S2. Proportion of LMS-PAHs and HMW-PAHs in the different sections (US1: Upstream 1, US2: Upstream 2, MS: Middle Stream, DS: Downstream); data from Site 8 were not included due to the extremely high concentration.
